# Death Anxiety, Spiritual Well-Being, and Death Literacy Among Relatives of Patients Receiving Palliative Care in Türkiye: A Cross-Sectional Study

**DOI:** 10.3390/healthcare14121745

**Published:** 2026-06-17

**Authors:** Nermin Yılmaz, Havva Akpınar

**Affiliations:** 1 Department of Psychiatric Nursing, Institute of Health Sciences, Mugla Sıtkı Kocman University, Mugla 48000, Türkiye; nermiiingunay@icloud.com; 2 Department of Psychiatric Nursing, Faculty of Health Sciences, Mugla Sıtkı Kocman University, Mugla 48000, Türkiye

**Keywords:** palliative care, death anxiety, spiritual well-being, death literacy, family caregivers, holistic care

## Abstract

**Highlights:**

**What are the main findings?**

- Relatives of patients receiving palliative care were found to have relatively elevated death anxiety scores, comparatively higher spiritual well-being scores, and moderate death literacy scores.
- Higher death literacy among relatives of palliative care patients was associated with lower death anxiety, and spiritual well-being showed a positive correlation with both death anxiety and death literacy.

**What are the implications of the main findings?**

- Interventions related to the dying process, spiritual well-being, and reducing death anxiety can help caregivers cope with end-of-life anxieties.
- Holistic palliative care services should include interventions that involve multidisciplinary psychosocial and spiritual support for the relatives of patients receiving palliative care.

**Abstract:**

**Background**: Understanding the psychological and spiritual needs of family caregivers, including their experiences of death anxiety and levels of death literacy, is essential for delivering holistic palliative care. This study aimed to examine the relationships between death anxiety, spiritual well-being, and death literacy among relatives of patients receiving palliative care in Türkiye. **Methods**: A cross-sectional correlational study was conducted with 160 relatives of patients receiving treatment in a palliative care unit in Türkiye. The participants had a mean age of 47.56 ± 12.33 years, and 62.5% were male. Data were obtained using the Abdel-Khalek Death Anxiety Scale (ASDA), the Three-Factor Spiritual Well-Being Scale (SWBS), and the Death Literacy Index (DLI). **Results**: The mean scores were 61.34 ± 17.45 for ASDA, 122.94 ± 15.84 for SWBS, and 96.13 ± 16.36 for DLI. Participants exhibited relatively elevated death anxiety scores, comparatively higher spiritual well-being scores, and moderate death literacy scores within the study sample. Correlation analyses showed that death anxiety was positively associated with spiritual well-being and negatively associated with death literacy, whereas death literacy was positively associated with spiritual well-being. Regression analyses further indicated that spiritual well-being was independently and positively associated with both death anxiety and death literacy, whereas death literacy was independently and negatively associated with death anxiety. **Conclusions**: The findings suggest that psychosocial, spiritual, and informational dimensions should be considered together in holistic palliative care. Supporting death literacy and spiritual well-being may contribute to better coping with death-related concerns among relatives of patients receiving palliative care.

## 1. Introduction

According to the World Health Organization (WHO), palliative care is a holistic and patient-centered approach that seeks to enhance the well-being of individuals with life-threatening illnesses and their families. It focuses on the early recognition, assessment, and management of physical, psychosocial, and spiritual challenges associated with serious illness. Palliative care also supports individuals and their relatives throughout the disease trajectory by promoting comfort, dignity, and quality of life through interdisciplinary care [1,2].

Palliative care extends beyond symptom management to encompass the holistic consideration of individuals’ biopsychosocial and spiritual needs [3,4]. Within this framework, key components include symptom control, spiritual care, and a holistic approach that addresses the biological, psychological, social, and existential dimensions of individuals. In particular, addressing the multidimensional needs of both patients and their families is fundamental to ensuring effective and compassionate care [5,6].

Family caregivers of patients receiving palliative care often experience significant emotional, psychological, and existential challenges as they witness disease progression and end-of-life processes. In this context, meeting caregivers’ spiritual needs is considered a core element of holistic care [7]. Spiritual care can facilitate meaning-making, strengthen coping capacities, and support caregivers in managing uncertainty and distress associated with illness and impending loss [8]. Spirituality is generally defined as an individual’s search for meaning, purpose, connection, and transcendence, which may or may not be expressed through religious beliefs and practices. It is recognized as an important internal resource that supports individuals in coping with adversity by fostering hope, resilience, meaning, and a sense of purpose in life [7,9]. Spiritual well-being reflects the extent to which individuals experience meaning, inner harmony, and connectedness with themselves, others, nature, or a transcendent reality. Particularly in life-threatening conditions and end-of-life contexts, spiritual needs become more prominent and may contribute to improved psychological adjustment and reduced anxiety [10,11].

Family caregivers in palliative care settings are frequently exposed to multiple stressors, including uncertainty about the future, caregiving burden, emotional distress, and anticipatory grief. Among these challenges, death anxiety represents a significant psychological concern [12,13,14]. Elevated levels of death anxiety may negatively affect caregivers’ communication, caregiving performance, and ability to provide emotional support [15]. Furthermore, caregivers are at increased risk of developing complicated grief and depressive symptoms, highlighting the importance of early assessment and intervention [16].

International studies have increasingly emphasized the importance of psychosocial, spiritual, and informational resources in end-of-life care. Family caregivers and healthcare professionals frequently experience emotional distress, uncertainty, and challenges related to coping with illness progression and impending loss. Spiritual coping, meaning-making, and adequate knowledge regarding end-of-life care have been identified as important factors that may facilitate adaptation and improve the quality of care experiences. In addition, greater knowledge of palliative and end-of-life care has been associated with more positive attitudes toward care provision and increased preparedness for death-related processes [3,13]. Collectively, these findings highlight the importance of examining psychological, spiritual, and cognitive dimensions simultaneously within palliative care settings.

In recent years, death literacy has gained increasing attention in palliative care research. The concept encompasses individuals’ knowledge, understanding, and practical abilities related to end-of-life care processes and available care options [17]. Higher levels of death literacy may support informed decision-making, improve communication about death and dying, and facilitate coping during end-of-life experiences. It promotes informed decision-making, enhances engagement in caregiving roles, and reduces fear and avoidance related to death [12]. Higher levels of death literacy are associated with improved access to care, better coping with end-of-life situations, and greater participation in care processes [18,19].

Within palliative care settings, spiritual well-being may be conceptualized as an adaptive psychological resource that helps individuals make meaning of illness, suffering, and mortality. Relatives with higher levels of spiritual well-being may be better able to interpret end-of-life experiences within a broader existential framework, potentially influencing how they perceive and respond to death-related concerns. Similarly, death literacy may facilitate understanding of dying processes and end-of-life care, thereby reducing uncertainty and supporting adaptive coping. From this perspective, spiritual well-being and death literacy may represent interconnected resources that shape caregivers’ responses to death-related experiences and may be associated with variations in death anxiety. However, the specific mechanisms linking these constructs remain unclear and require further investigation using longitudinal and theory-driven research designs.

From a holistic care perspective, addressing psychological (death anxiety), spiritual (spiritual well-being), and cognitive (death literacy) dimensions simultaneously is essential for the comprehensive assessment and support of family caregivers. Examining these interrelated constructs together may provide a more comprehensive understanding of caregivers’ psychosocial and spiritual experiences and may inform the development of holistic palliative care practices. Although research addressing the psychological and spiritual dimensions of palliative care has increased in recent years, studies simultaneously examining death anxiety, spiritual well-being, and death literacy among relatives of patients receiving palliative care remain limited. Furthermore, the relationships among these variables have not been sufficiently explored within a holistic care framework. Accordingly, the present study aimed to examine the levels of death anxiety, spiritual well-being, and death literacy among relatives of patients receiving palliative care and to investigate the relationships among these variables. To address this aim, the following research questions were formulated:

1. What are the levels of death anxiety, spiritual well-being, and death literacy among relatives of patients hospitalized in a palliative care unit?
2. Are death anxiety, spiritual well-being, and death literacy influenced by participants’ sociodemographic and caregiving-related characteristics?
3. Is there a significant relationship between death anxiety, spiritual well-being, and death literacy among relatives of patients receiving palliative care?
4. To what extent are death anxiety, spiritual well-being, and death literacy independently associated with one another among relatives of patients receiving palliative care?

## 2. Materials and Methods

### 2.1. Study Design

This research employed a cross-sectional, descriptive, and correlational design and was reported in line with the Strengthening the Reporting of Observational Studies in Epidemiology (STROBE) guidelines [20].

### 2.2. Participants and Procedure

The study population consisted of relatives of patients receiving treatment in the palliative care unit of a training and research hospital in Türkiye. During the study period, 225 patients were receiving care in the unit, and their eligible relatives were invited to participate in the study (N = 225). Sample size estimation was performed using G*Power software version 3.1.9.7 (Heinrich Heine University Düsseldorf, Düsseldorf, Germany) [21]. The estimation was based on examining the relationships among the three scales used in the study, namely, the Abdel-Khalek Death Anxiety Scale (ASDA), Three-Factor Spiritual Well-Being Scale (SWBS), and Death Literacy Index (DLI), using multiple linear regression analysis. In each model, one scale was treated as the dependent variable, while the other two were considered predictor variables. Based on Cohen’s effect size classification, a medium effect size (f^2^ = 0.15) was assumed, with the significance level set at α = 0.05 and statistical power at 0.95 [22]. Considering that each regression model included two predictor variables, an a priori power analysis was performed using the “Linear multiple regression: Fixed model, R^2^ deviation from zero” procedure in GPower. The analysis indicated that at least 107 participants were required to achieve adequate statistical power [21]. To account for potential data loss, the required sample size was increased by 20% [23], resulting in a minimum target of 128 participants. A convenience sampling approach was used, and all eligible relatives present during the study period were invited to participate in the study. The final sample included 160 relatives of patients receiving treatment in the palliative care unit who met the inclusion criteria, voluntarily agreed to participate, and completed the data collection forms without missing data (n = 160). Individuals who declined participation, did not meet the eligibility criteria, withdrew after providing consent, or submitted incomplete questionnaires were not included in the final sample. Multiple relatives of the same patient were included in the study, provided they met the participation criteria and voluntarily agreed to participate. Because the study focused on the individual experiences and perceptions of relatives regarding death anxiety, spiritual well-being, and death literacy, rather than patient-related outcomes, each participant was considered an independent unit of analysis and completed the questionnaires individually.

#### Inclusion and Exclusion Criteria

Participants were eligible for inclusion if they voluntarily agreed to participate in the study, were able to read and write, and completed the data collection forms fully and accurately. Individuals were excluded if they declined participation, withdrew from the study after providing consent, were unable to read or write, or submitted incomplete or inaccurately completed data collection forms.

### 2.3. Data Collection Instruments

Data was collected using the Descriptive Information Form, the Abdel-Khalek Death Anxiety Scale (ASDA), the Three-Factor Spiritual Well-Being Scale (SWBS), and the Death Literacy Index (DLI).

#### 2.3.1. Descriptive Information Form

The Descriptive Information Form was prepared by the researchers in accordance with the relevant literature [6,7,9,12,14]. It consists of eight questions addressing participants’ age, gender, educational status, relationship to the patient, duration of caregiving, as well as the patient’s age and duration of treatment in the palliative care unit. Gender was assessed using two response categories, female and male.

#### 2.3.2. Abdel-Khalek Death Anxiety Scale

The Abdel-Khalek Death Anxiety Scale was originally developed by Abdel-Khalek (2004) [24], and its Turkish validity and reliability were established by Aydoğan et al. (2015) [25]. The instrument includes 20 items scored on a five-point Likert scale ranging from 1 (“not at all”) to 5 (“very much”). The scale does not contain reverse-coded items. Total scores range between 20 and 100, with higher scores reflecting greater levels of death anxiety. The Cronbach’s alpha coefficient for the Turkish version was reported as 0.86 [25], whereas the coefficient calculated in the present study was 0.94.

#### 2.3.3. Three-Factor Spiritual Well-Being Scale

The Three-Factor Spiritual Well-Being Scale was developed by Ekşi and Kardaş (2017) and Kardaş (2019) [26,27]. The scale consists of 29 items rated on a five-point Likert-type scale from 1 (“not appropriate at all”) to 5 (“completely appropriate”). Possible total scores range from 29 to 145, with higher scores indicating greater spiritual well-being. Previous studies reported Cronbach’s alpha coefficient of 0.88 for the original scale [26,27], while the coefficient obtained in the present study was 0.92. The scale was selected because it was developed and validated in the Turkish cultural context and provides a multidimensional assessment of spiritual well-being.

#### 2.3.4. Death Literacy Index

The Death Literacy Index was developed by Leonard et al. (2021) [28], and the Turkish validity and reliability study was conducted by Semerci, Sönmez, and Seven (2024) [17]. The scale contains 29 items rated on five-point Likert-type response formats. Depending on the item content, response options range from 1 (e.g., very difficult) to 5 (e.g., very easy). Total scores range from 29 to 145, with higher scores indicating greater levels of death literacy. The Cronbach’s alpha coefficient for the Turkish version was reported as 0.90 [17], whereas the coefficient calculated in this study was 0.91.

### 2.4. Data Collection

Data were collected between 15 January and 31 March 2025 from relatives of patients receiving treatment in the palliative care unit of a training and research hospital in Türkiye. Face-to-face data collection was conducted by the researcher in a private interview room within the unit at times appropriate for the participants. All procedures were performed in accordance with hospital regulations, ethical principles, infection control measures, and patient rights, while care was taken not to interfere with patient care or clinical workflow. Participants who voluntarily agreed to participate were provided with the data collection forms, which took approximately 10 min to complete.

### 2.5. Statistical Analysis

Statistical analyses were conducted using IBM SPSS Statistics version 27.0 (IBM Corp., Armonk, NY, USA). Descriptive statistics were used to summarize the data. Categorical variables were presented as frequencies and percentages, whereas continuous variables were expressed as means and standard deviations.

Before conducting inferential analyses, the suitability of the data for parametric testing was evaluated. Normality was assessed using histograms, skewness and kurtosis values, and the Kolmogorov–Smirnov test. Since the skewness and kurtosis z-values exceeded ±1.96 and the Kolmogorov–Smirnov test yielded significant results (*p* < 0.05), the scale scores were considered non-normally distributed [29]. Therefore, nonparametric methods were preferred for group comparisons. To facilitate interpretation and reduce analytical complexity, analyses were conducted using the total scores of the scales.

Comparisons between two independent groups were performed using the Mann–Whitney U test, whereas differences among three or more groups were examined using the Kruskal–Wallis H test. When the Kruskal–Wallis H test indicated statistically significant overall group differences, post hoc analyses were conducted to identify pairwise differences between groups. In cases where variance homogeneity was not satisfied, Tamhane’s T2 procedure was applied as a conservative multiple-comparison approach [30,31].

Relationships among the study variables were examined using Spearman’s rank correlation coefficient. In addition, multiple linear regression analyses were conducted to examine the independent associations among death anxiety, spiritual well-being, and death literacy. In each regression model, one variable was treated as the dependent variable, and the remaining variables were entered as independent variables. Prior to the regression analyses, assumptions related to linearity, independence of errors, multicollinearity, and residual distributions were evaluated. Independence of errors was assessed using the Durbin–Watson statistic, which yielded values ranging from 1.38 to 1.66 across the models, indicating acceptable independence of residuals. Multicollinearity was evaluated using tolerance and variance inflation factor (VIF) values. Tolerance values ranged from 0.938 to 1.000, and VIF values ranged from 1.000 to 1.067, indicating no evidence of multicollinearity. Standardized residuals were also examined, and no substantial outliers were identified. These diagnostic results supported the suitability of the regression analyses. Reliability analyses were conducted using Cronbach’s alpha coefficients. Statistical significance was set at *p* < 0.05 with a 95% confidence level [30,32]. To obtain a more concise interpretation and reduce analytical complexity, analyses were performed using only the total scores of the scales. Although all three instruments include multiple subdimensions, analyses were conducted using only the total scale scores, consistent with the study objectives and to facilitate the examination of the overall relationships among death anxiety, spiritual well-being, and death literacy.

### 2.6. Ethical Considerations

Ethical approval for the study was obtained from the Medical and Health Sciences Ethics Committee-2 of a public university (dated 4 November 2024; decision no: 240166-130). Institutional permission was also obtained from the hospital where the research was conducted, and permission for the use of the measurement tools was secured from the relevant authors.

Participation in the study was entirely voluntary. Before data collection, participants were informed about the purpose and procedures of the study through an informed consent form. Only individuals who agreed to participate were included in the study. Participants were informed that no identifying information would be collected, that they could withdraw from the study at any stage without any consequences, and that all collected data would remain confidential. All participants were treated equally throughout the research process. The study was carried out in accordance with the ethical principles outlined in the Declaration of Helsinki (World Medical Association, 2024/75 General Assembly).

## 3. Results

Table 1 summarizes the descriptive characteristics of the participants and the patients receiving palliative care. The mean age of the participants was 47.56 ± 12.33 years (range: 19–71 years), and half of them were between 39 and 58 years of age. Most participants were women and married. Regarding educational background, primary/secondary school graduates constituted the largest group, followed by university graduates. In terms of relationships with the patient, mothers, fathers, and spouses represented the majority of participants. More than half of the participants had been providing care for longer than three months. The mean age of the patients was 70.44 ± 14.09 years (range: 19–101 years), and most patients were aged 69 years or older. Additionally, a considerable proportion of the patients had been receiving treatment in the palliative care unit for more than one month.

Table 2 presents the mean scale scores of the participants. The mean ASDA score was 61.34 ± 17.45, the mean SWBS score was 122.94 ± 15.84, and the mean DLI score was 96.13 ± 16.36.

Table 3 shows the differences in death anxiety, spiritual well-being, and death literacy scores according to participants’ descriptive characteristics. Female participants had significantly higher death anxiety scores than male participants (*p* = 0.001). Similarly, married participants reported higher death anxiety scores compared with single participants (*p* = 0.004). Participants with primary/secondary school education had significantly higher death anxiety and spiritual well-being scores than those with higher educational levels (*p* < 0.05). Regarding relationship to the patient, participants caring for their spouses had significantly higher death anxiety scores than those classified in the “other” category (*p* = 0.007). In addition, participants caring for their mothers had significantly higher death literacy scores compared with the “other” group (*p* = 0.024). Participants caring for younger patients (19–48 years) had significantly higher spiritual well-being and death literacy scores than those caring for patients aged 69 years and older (*p* < 0.05). No significant differences were identified in death anxiety, spiritual well-being, or death literacy according to caregiving duration or the length of the patient’s treatment in the palliative care unit (*p* > 0.05).

Table 4 presents the relationships among death anxiety, spiritual well-being, and death literacy scores. Correlation analyses indicated a weak positive relationship between death anxiety and spiritual well-being (r = 0.250, *p* < 0.05). In contrast, death anxiety showed a weak negative association with death literacy (r = −0.160, *p* < 0.05). Additionally, spiritual well-being and death literacy were positively correlated at a weak level (r = 0.378, *p* < 0.05).

Variables with significant correlations were further examined using multiple linear regression analyses to investigate the independent relationships among the study variables (Table 5). The findings revealed reciprocal relationships between death anxiety, spiritual well-being, and death literacy. Regression analyses indicated that death literacy was independently and negatively associated with death anxiety (β = −0.303, *p* < 0.001), whereas spiritual well-being was independently and positively associated with death anxiety (β = 0.359, *p* < 0.001). Together, these variables accounted for 14.2% of the variance in death anxiety scores. In the second regression model, both death anxiety (β = 0.322, *p* < 0.001) and death literacy (β = 0.416, *p* < 0.001) were independently and positively associated with spiritual well-being, explaining 23.0% of the variance in spiritual well-being scores. In the final regression model, death anxiety was independently and negatively associated with death literacy (β = −0.281, *p* < 0.001), whereas spiritual well-being was independently and positively associated with death literacy (β = 0.430, *p* < 0.001). This model explained 20.4% of the variance in death literacy.

## 4. Discussion

In the present study, the relationships between death anxiety, spiritual well-being, and death literacy in relatives of patients receiving palliative care were explored and interpreted within the context of the relevant literature. The findings indicated that more than half of the participants had been caring for the patient for longer than three months, while nearly half of the patients had remained in the palliative care unit for more than one month. Comparable findings have been reported in previous studies [33,34,35], which also identified extended caregiving periods among relatives of patients receiving palliative care. Long-term caregiving is frequently encountered in palliative care settings because patients often require continuous physical, emotional, and caregiver support [36]. Extended caregiving responsibilities may increase caregivers’ emotional and psychological burden and make coping with illness progression and end-of-life concerns more difficult. These findings emphasize the importance of providing psychosocial and supportive care for relatives involved in long-term caregiving processes.

In the present study, the average age of patients receiving palliative care was approximately 70 years, and nearly all patients were older than 50 years of age. Similar findings have been reported in previous studies, indicating that the majority of patients receiving palliative care are older adults. Similarly, Aksüt (2024) reported that all patients in the palliative care unit were older than 60 years [33], while Yürüyen et al. (2018) found a mean patient age of 71 years [37]. The increasing life expectancy, growth of the elderly population, advances in medical treatment opportunities, and improved access to healthcare services have contributed to the increasing number of older adults receiving care in palliative care clinics [38]. The findings of the present study support previous research and may reflect the increasing demand for long-term palliative and caregiver support among older adults with chronic and progressive illnesses. These results also indicate that the need for palliative care services and the number of individuals requiring care in palliative care units may continue to rise in the future.

Based on the mean ASDA scores, relatives of patients receiving palliative care exhibited relatively elevated death anxiety scores within the study sample. Death anxiety is generally described as an emotional response associated with awareness of mortality, uncertainty, loss, and concerns related to nonexistence [39]. Previous studies involving relatives of patients receiving intensive and palliative care have similarly reported moderate to high levels of death anxiety among caregivers [34,40,41]. The findings of the present study are therefore compatible with the existing literature and suggest that relatives involved in palliative caregiving may be psychologically affected by prolonged exposure to illness and end-of-life processes.

In the present study, women reported higher levels of death anxiety than men. Similar findings have been identified in earlier studies, suggesting that female participants tend to experience greater death-related anxiety compared with male participants [39,42,43]. Other studies conducted with relatives of patients receiving palliative care have also demonstrated higher death anxiety levels among women [36,41]. These findings suggest that gender may be an important determinant of death anxiety.

According to the findings of the present study, married participants had higher death anxiety levels than single participants. Similarly, Görücü and Arslan (2024) reported higher death anxiety levels among married individuals [41]. However, some studies have reported contradictory findings, including lower death anxiety levels among married individuals or no significant relationship between marital status and death anxiety [44,45]. These inconsistencies may be related to sociocultural factors, individual living conditions, and differences in sample characteristics.

In the present study, participants caring for their spouses had higher death anxiety levels than those caring for individuals classified in the “other (child, sibling, spouse’s mother/father, aunt, or uncle)” category. Similarly, caregivers of patients receiving palliative care may experience considerable psychological burden because of frequent exposure to death-related experiences and the chronic stress associated with the caregiving process [40]. Previous studies have shown that caregiving burden and emotional exhaustion are high among relatives of patients receiving palliative care [46]. Particularly during the end-of-life period, relatives accompanying patients may experience high levels of uncertainty, fear, and anxiety [36].

In the present study, spiritual well-being among relatives of patients receiving palliative care was assessed using the SWBS, and participants exhibited relatively higher spiritual well-being scores within the study sample. Spirituality represents an important dimension influenced by individuals’ life experiences and may play a significant role in the caregiving process for patients receiving palliative care. Spiritual beliefs and practices may affect coping abilities, emotional responses, and adaptation to illness-related challenges [47]. Similar findings have been reported in previous studies. Görücü and Gürol Arslan (2024) identified high spiritual well-being levels among relatives of patients receiving intensive care treatment [41]. Other studies have likewise demonstrated elevated levels of spirituality and spiritual well-being among caregivers [35,47,48]. These findings suggest that spirituality may support caregivers in coping with emotional and existential difficulties encountered during the caregiving and end-of-life process.

Meeting the spiritual needs of patients and their relatives is considered one of the essential components of holistic healthcare. Particularly during the end-of-life period, spirituality, hope, meaning-making, acceptance, and dignity become increasingly important for both patients and their relatives. Approaches incorporating spiritual care may contribute to improving quality of life, reducing suffering, and supporting coping during the palliative care process [7]. In addition, receiving spiritual care during this challenging period may help relatives of patients receiving palliative care better understand uncertainties, make sense of positive and negative experiences, strengthen problem-solving skills, and establish more effective caregiving relationships [8].

In the present study, death literacy levels among relatives of patients receiving palliative care were evaluated using the DLI, and participants exhibited relatively elevated death literacy scores within the study sample. Because death literacy is a relatively recent concept in the literature, studies examining this topic remain limited. A community-based study conducted in the United Kingdom reported moderate levels of death literacy among participants [49]. Similarly, a study conducted with Swedish adults found moderate levels of death literacy among participants [18]. In Türkiye, a study involving patients with chronic illnesses reported that death literacy levels were slightly above average [39]. The findings of the present study are consistent with the existing literature.

Death literacy is a multidimensional concept encompassing the knowledge and skills required to understand, access, and make informed decisions regarding end-of-life care [18,28]. Individuals with higher levels of death literacy are better prepared to understand, access, and utilize end-of-life care resources and services effectively [49]. In palliative care, death literacy has been recognized as an important component of informed decision-making and community capacity for end-of-life support. Moreover, increasing life expectancy and the growing burden of chronic diseases further emphasize the need to strengthen health literacy and supportive care services, including palliative care [50].

In the present study, death anxiety was positively associated with spiritual well-being among relatives of patients receiving palliative care. Regression analyses also indicated that spiritual well-being was significantly associated with death anxiety, with higher spiritual well-being scores accompanying higher death anxiety scores. Similar findings were reported by Görücü and Gürol Arslan (2024), who identified a positive relationship between spiritual well-being and death anxiety among relatives of patients receiving intensive care treatment [41]. Thus, the findings of the present study are consistent with some previous research.

However, the literature presents mixed findings regarding the relationship between spiritual well-being and death anxiety. For example, Moetamedi et al. (2015) reported that spirituality may strengthen positive mental processes, hope, and emotional resilience in coping with death-related anxiety and suggested that individuals may increasingly turn to spiritual values and resources when experiencing heightened death anxiety [51]. Similarly, Solaimanizadeh et al. (2019) found that death anxiety decreased as spiritual well-being increased [52]. MacLeod et al. (2019) also reported that individuals with higher levels of spiritual well-being were better able to cope with anxiety related to actual or anticipated death [53].

The findings should also be interpreted within the sociocultural context of Türkiye. Family members traditionally play a central role in caregiving and decision-making for individuals with serious illnesses, particularly during end-of-life care. Strong family ties may increase emotional involvement in the caregiving process and intensify concerns related to illness progression, suffering, and death. At the same time, religious and spiritual beliefs often serve as important sources of meaning, comfort, and support when individuals are confronted with mortality. These cultural, familial, and spiritual factors may contribute to the complex relationships observed among death anxiety, spiritual well-being, and death literacy in the present study.

Accordingly, the positive association observed between spiritual well-being and death anxiety should be interpreted with caution. Given the cross-sectional design, the direction and underlying mechanisms of this relationship cannot be determined. Although spiritual well-being is frequently regarded as a protective resource against existential distress, it is also possible that heightened awareness of mortality and death-related concerns encourages greater existential reflection, meaning-making, and engagement with spiritual beliefs and practices. Therefore, the observed association may reflect complex and potentially reciprocal processes shaped not only by individual factors but also by broader cultural and caregiving-related influences. Future longitudinal studies employing mediation and moderation analyses are needed to clarify the temporal, causal, and interactive nature of these relationships among relatives of patients receiving palliative care.

A negative relationship was found between death anxiety and death literacy among relatives of patients receiving palliative care. Furthermore, regression analyses indicated that death literacy was independently and negatively associated with death anxiety, suggesting that higher death literacy scores were accompanied by lower death anxiety scores. Similarly, death anxiety was independently and negatively associated with death literacy, indicating an inverse relationship between these variables. Given the cross-sectional nature of the study, the direction of these associations cannot be determined. These findings suggest a reciprocal negative association between death anxiety and death literacy. Supporting this result, Zhang et al. (2024) identified an inverse relationship between death literacy and death anxiety and reported that higher levels of death literacy were associated with reduced death anxiety [54]. Similarly, Semerci Çakmak, Seven, and Sönmez Sarı (2025), in a study involving individuals with chronic illnesses, found that death literacy was negatively correlated with death anxiety and suggested that increasing death literacy may help alleviate death-related anxiety [39]. Overall, the findings of the present study are compatible with the existing literature.

Death literacy encompasses the knowledge, skills, and awareness required to understand death-related processes, navigate end-of-life care, and support individuals during the dying process [18]. Individuals with higher levels of death literacy are generally better equipped to access and utilize end-of-life care resources and supportive services [19]. Conversely, limited knowledge about death and end-of-life care may contribute to uncertainty and death-related concerns. By improving understanding of death-related processes and facilitating informed engagement with end-of-life care, death literacy may support adaptation to end-of-life experiences [28,39].

In the present study, death literacy was positively associated with spiritual well-being among relatives of patients receiving palliative care. Regression analyses further indicated an independent positive association between death literacy and spiritual well-being. However, given the cross-sectional nature of the study, the direction of this relationship cannot be determined. Because death literacy is a relatively recent concept, studies exploring its relationship with spiritual well-being remain scarce. No previous study directly examining this relationship among relatives of patients receiving palliative care was identified.

Nevertheless, Görücü and Gürol Arslan (2024) reported a positive association between spiritual well-being and death anxiety among relatives of patients receiving intensive care treatment [41]. Similarly, Leonard et al. (2021) identified a positive relationship between spiritual beliefs and death literacy [28], supporting the findings of the present study. Relatives of patients receiving palliative care are frequently confronted with end-of-life experiences, and adequate knowledge and skills regarding death-related processes may enhance their ability to adapt to the challenges associated with caregiving and impending loss. From a comprehensive care perspective, addressing the psychosocial, spiritual, and informational needs of both patients and their relatives is an essential component of palliative care. In this context, death literacy and spiritual well-being may facilitate adaptation to end-of-life experiences and contribute to high-quality palliative care. Furthermore, death literacy has been recognized as a key component of community capacity for end-of-life care and support [18]. Therefore, higher levels of death literacy and spiritual well-being may help reduce death-related distress and support adaptation during the caregiving process.

Although the regression models were statistically significant, the proportion of explained variance was modest (R^2^ = 14.2–23.0%). These findings suggest that death anxiety, spiritual well-being, and death literacy are influenced by multiple factors beyond those examined in the present study. Other psychosocial, demographic, cultural, clinical, and caregiving-related variables may contribute to these outcomes and should be considered in future research. Therefore, the observed associations should be interpreted within the context of a broader set of determinants that were not assessed in the current study.

This study has several strengths. First, to our knowledge, it is among the first studies conducted in Türkiye to examine death anxiety, spiritual well-being, and death literacy simultaneously among relatives of patients receiving palliative care. By evaluating these psychological, spiritual, and cognitive dimensions together, the study provides a more comprehensive understanding of caregivers’ experiences within a holistic care framework. Second, the study contributes to the emerging literature on death literacy, a relatively new concept that has received limited attention in palliative care research. Third, the use of validated and reliable measurement instruments strengthened the methodological rigor of the study. Fourth, multiple regression analyses enabled the examination of independent associations among the study variables while accounting for their interrelationships. Finally, the focus on family caregivers directly involved in palliative care provides clinically relevant insights that may inform future research and supportive care practices.

This study has several limitations that should be considered when interpreting the findings. First, the study was conducted in a single palliative care unit using a convenience sampling approach, which may limit the generalizability of the findings and introduce selection bias. Second, the cross-sectional design allowed the variables to be assessed at only one point in time and therefore precludes conclusions regarding causality or the temporal direction of the observed associations. Third, all data were collected using self-report measures, which may be subject to response bias, common-method bias, and social desirability effects. This issue may be particularly relevant for spirituality-related constructs, as participants’ responses may have been influenced by social, cultural, or personal expectations.

In addition, several potentially important factors were not assessed in the present study, including psychiatric history, religious affiliation, caregiver burden, socioeconomic status, previous bereavement experiences, and formal psychiatric screening. Furthermore, patient-related clinical characteristics, such as disease severity, prognosis, functional status, and symptom burden, were not evaluated. These factors may have influenced relatives’ experiences of death anxiety, spiritual well-being, and death literacy and should therefore be considered when interpreting the findings.

Finally, although statistically significant associations were identified, the regression models explained a relatively modest proportion of the variance in the study variables, suggesting that additional psychosocial, cultural, clinical, and caregiving-related factors may contribute to these outcomes. Because the study was conducted in Türkiye, where cultural, religious, and family-related factors may shape experiences of death, spirituality, and caregiving, the findings should be generalized to other cultural contexts with caution. Future longitudinal and multicenter studies incorporating a broader range of contextual and clinical variables are needed to provide a more comprehensive understanding of the relationships among death anxiety, spiritual well-being, and death literacy among relatives of patients receiving palliative care.

## 5. Conclusions

This study examined the relationships among death anxiety, spiritual well-being, and death literacy in relatives of patients receiving palliative care in Türkiye. The findings indicated relatively elevated death anxiety and spiritual well-being scores, while death literacy scores were moderate within the study sample. Significant associations among these variables suggested that they represent interconnected psychosocial and spiritual dimensions of the caregiving experience. Death literacy showed a negative association with death anxiety, whereas spiritual well-being showed positive associations with both death anxiety and death literacy. Furthermore, higher spiritual well-being scores were associated with higher death literacy scores, while higher death literacy scores were associated with lower death anxiety scores.

These findings contribute to a better understanding of the relationships among death anxiety, spiritual well-being, and death literacy among relatives of patients receiving palliative care. However, given the cross-sectional design of the study, the direction and underlying mechanisms of these associations cannot be determined and should therefore be interpreted with caution. The findings highlight the relevance of psychological, spiritual, and informational factors within the caregiving experience and support the importance of considering these dimensions within palliative care. Future qualitative, longitudinal, experimental, multicenter, and cross-cultural studies are needed to clarify the mechanisms underlying these associations and to examine whether similar patterns are observed across different populations, cultural contexts, and caregiving groups.

## Figures and Tables

**Table 1:** Distribution of participants according to descriptive characteristics.

Descriptive Characteristics	n	%
*Characteristics of Patients’ Relatives*		
Age (years) $\bar{X}$ = 47.56 ± 12.33 (Min = 19, Max = 71)		
19–38	44	27.50
39–58	80	50.00
≥59	36	22.50
Gender		
Female	60	37.50
Male	100	62.50
Marital Status		
Married	126	78.75
Single	34	21.25
Educational Status		
Primary/Secondary School	66	41.25
High School	37	23.13
University	57	35.62
Patient’s Relationship to the Participant		
Mother	45	28.13
Father	43	26.88
Spouse	30	18.75
Other *	42	26.24
Duration of Caregiving for the Patient in the Palliative Care Unit		
0–30 days	36	22.50
1–3 months	42	26.25
>3 months	82	51.25
*Characteristics of Patients Receiving Palliative Care*		
Patient Age (years) $\bar{X}$ = 70.44 ± 14.09 (Min = 19, Max = 101)		
19–48	8	5.00
49–68	45	37.50
≥69	65	57.50
Duration of Treatment in the Palliative Care Unit		
1–7 days	50	31.25
8–30 days	45	28.13
>1 month	65	40.62
Total	160	100

Note: * Other: Relatives included children, siblings, and spouse’s parents, aunts, and uncles.

**Table 2:** Mean total scores of the scales among patients’ relatives.

Scales (n = 160)	Min	Max	$\bar{X}$ ± SD
Abdel-Khalek Death Anxiety Scale (ASDA)	20	100	61.34 ± 17.45
Three-Factor Spiritual Well-Being Scale (SWBS)	79	144	122.94 ± 15.84
Death Literacy Index (DLI)	37	138	96.13 ± 16.36

Notes: ASDA: Abdel-Khalek Death Anxiety Scale; SWBS: Spiritual Well-Being Scale; DLI: Death Literacy Index; SD: Standard Deviation.

**Table 3:** Differences in ASDA, SWBS, and DLI scores by descriptive characteristics of patients’ relatives.

Descriptive Characteristics	ASDA Mean ± SD Test and *p*	SWBS Mean ± SD Test and *p*	DLI Mean ± SD Test and *p*
Gender			
Female	3.27 ± 0.82	4.30 ± 0.52	3.22 ± 0.54
Male	2.72 ± 0.84	4.14 ± 0.57	3.38 ± 0.56
	U = 1834.00, *p* = 0.001	U = 2494.50, *p* = 0.075	U = 3465.00, *p* = 0.101
Marital Status			
Married	3.17 ± 0.80	4.26 ± 0.53	3.25 ± 0.53
Single	2.68 ± 1.02	4.17 ± 0.61	3.37 ± 0.60
	U = 1452.00, *p* = 0.004	U = 1983.50, *p* = 0.508	U = 2382.00, *p* = 0.317
Educational Status			
Primary/Secondary School ^1^	3.48 ± 0.69	4.40 ± 0.45	3.32 ± 0.38
High School ^2^	2.92 ± 0.72	4.09 ± 0.55	3.19 ± 0.68
University ^3^	2.69 ± 0.95	4.15 ± 0.60	3.30 ± 0.62
	KW = 33.11, *p* = 0.001	KW = 10.413, *p* = 0.005	KW = 0.556, *p* = 0.753
Post hoc=	1 > 2, 3	1 > 2, 3	
Degree of Relationship to the Patient			
Mother ^1^	2.99 ± 0.94	4.16 ± 0.61	3.43 ± 0.58
Father ^2^	2.98 ± 0.75	4.32 ± 0.53	3.03 ± 0.39
Spouse ^3^	3.49 ± 0.58	4.46 ± 0.46	3.17 ± 0.31
Other ^4^	2.93 ± 1.01	4.09 ± 0.50	3.15 ± 0.72
	KW = 12.007, *p* = 0.007	KW = 3.105, *p* = 0.376	KW = 9.421, *p* = 0.024
Post hoc=	3 > 4		1 > 4
Patient Age Group			
19–48	2.91 ± 1.34	4.37 ± 0.63	3.57 ± 0.90
49–68	3.21 ± 0.68	4.16 ± 0.50	3.39 ± 0.42
≥69	2.98 ± 0.93	4.03 ± 0.56	3.19 ± 0.58
	KW = 2.864, *p* = 0.239	KW = 6.873, *p* = 0.032	KW = 5.716, *p* = 0.005
Post hoc=		1 > 3	1 > 3
Duration of Caregiving			
0–30 days	2.99 ± 0.98	4.16 ± 0.58	3.25 ± 0.68
1–3 months	3.24 ± 0.72	4.27 ± 0.56	3.29 ± 0.40
>3 months	3.01 ± 0.88	4.26 ± 0.53	3.29 ± 0.56
	KW = 1.458, *p* = 0.482	KW = 1.103, *p* = 0.576	KW = 0.096, *p* = 0.953
Duration of Patient Treatment in the Palliative Care Unit			
1–7 days	3.06 ± 0.91	4.19 ± 0.57	3.23 ± 0.64
8–30 days	3.05 ± 0.74	4.29 ± 0.50	3.29 ± 0.47
>1 month	3.08 ± 0.93	4.24 ± 0.56	3.30 ± 0.53
	KW = 0.373, *p* = 0.830	KW = 0.872, *p* = 0.647	KW = 0.420, *p* = 0.811

Notes: SD: Standard deviation; U: Mann–Whitney U test; KW: Kruskal–Wallis test; Post hoc: Tamhane’s T2 test; Superscript numbers (1–4) correspond to the group numbers presented in the first column and indicate significant pairwise differences; ASDA: Abdel-Khalek Death Anxiety Scale; SWBS: Spiritual Well-Being Scale; DLI: Death Literacy Index; Other: Relatives included children, siblings, spouse’s parents, aunts, and uncles.

**Table 4:** Correlations between the mean total scores of the scales among relatives of patients.

Scales	1	2	3
1. Abdel-Khalek Death Anxiety Scale (ASDA)	1		
2. Spiritual Well-Being Scale (SWBS)	0.250 *	1	
3. Death Literacy Index (DLI)	−0.160 *	0.378 *	1

Notes: Spearman correlation analysis; * *p* < 0.05.

**Table 5:** Multivariate regression models examining the relationships between death anxiety, spiritual well-being, and death literacy.

Predictor	Model 1: ASDA β	*p*	Model 2: SWBS β	*p*	Model 3: DLI β	*p*
ASDA	-	-	0.322	<0.001	−0.281	<0.001
SWBS	0.359	<0.001	-	-	0.430	<0.001
DLI	−0.303	<0.001	0.416	<0.001	-	-
R^2^	0.142	-	0.230	-	0.204	-
F	13.040	<0.001	23.474	<0.001	20.088	<0.001
Durbin–Watson	1.59	-	1.38	-	1.66	-

Notes: ASDA = Abdel-Khalek Death Anxiety Scale; SWBS = Spiritual Well-Being Scale; DLI = Death Literacy Index; β = standardized regression coefficient. Prior to regression analyses, assumptions of independence of errors and multicollinearity were evaluated. Durbin–Watson statistics ranged from 1.38 to 1.66, indicating acceptable independence of errors. No evidence of multicollinearity was detected (tolerance = 0.938–1.000; VIF = 1.000–1.067).

## Data Availability

The data presented in this study is available on request from the corresponding author. The data is not publicly available due to privacy and ethical restrictions, as it contains sensitive personal and health-related information.

## Abbreviations

The following abbreviations are used in this manuscript:

WHO	World Health Organization
STROBE	Strengthening the Reporting of Observational Studies in Epidemiology
ASDA	Abdel-Khalek Death Anxiety Scale
SWBS	Three-Factor Spiritual Well-Being Scale
DLI	Death Literacy Index
